# Evaluation of the Characteristics of Digital Light Processing 3D-Printed Magnesium Calcium Phosphate for Bone Regeneration

**DOI:** 10.3390/jfb16040139

**Published:** 2025-04-14

**Authors:** Peng Zhang, Meiling Zhang, Yoo-Na Jung, Seong-Won Choi, Yong-Seok Lee, Geelsu Hwang, Kwi-Dug Yun

**Affiliations:** 1 Department of Prosthodontics, School of Dentistry, Chonnam National University, Gwangju 61186, Republic of Korea; zhangpeng8883@jnu.ac.kr (P.Z.); yoonajung@jnu.ac.kr (Y.-N.J.); 2 Department of Orthodontics, School of Dentistry, Chonnam National University, Gwangju 61186, Republic of Korea; 218451@jnu.ac.kr; 3 Industry Support Center for Convergence Medical Devices, Chonnam National University Hospital, Gwangju 61469, Republic of Korea; dg04300@cnuh.com; 4 Department of Mechanical Engineering, Myongji University, Yongin 17058, Republic of Korea; yslee23@mju.ac.kr; 5 Department of Preventive and Restorative Sciences, School of Dental Medicine, University of Pennsylvania, Philadelphia, PA 19104, USA; 6 Center for Innovation & Precision Dentistry, School of Dental Medicine, School of Engineering and Applied Sciences, University of Pennsylvania, Philadelphia, PA 19104, USA

**Keywords:** bone tissue engineering, DLP 3D printing, magnesium calcium phosphate, bone regeneration

## Abstract

Recent advancements in three-dimensional (3D) printing technology, particularly digital light processing (DLP) 3D printing, have enabled the customization of bone substitutes with specific shapes that match bone defect sizes and geometries. Magnesium calcium phosphate (MCP) has gained considerable attention due to its strong mechanical properties, degradability, and ability to promote bone regeneration. In this study, we prepared MCP samples with five different molar ratios via DLP 3D printing. We analyzed the physicochemical properties of these five groups, including phase compositions and microstructures, which were examined using X-ray diffraction and scanning electron microscopy, respectively. Additionally, we assessed the effects of MCP on material density and shrinkage. Biaxial flexural strength and degradation rate were evaluated; biological properties were examined through WST-8 analysis and alkaline phosphatase activity assays. Among the tested samples, MCP1/1 exhibited the highest strength. A higher proportion of magnesium phosphate in MCP corresponded to an increased degradation rate. Cell response observations in the WST-8 assay indicated that cell proliferation was better in the MCP1/1 group than in the other groups on days 4 and 7 of culturing. Alkaline phosphatase activity assays demonstrated that MCP1/1 exhibited higher activity than calcium phosphate. Our findings suggest that MCP1/1 can be used effectively in bone-tissue-engineering applications.

## 1. Introduction

Bone implant materials play a critical role in surgical procedures that repair bone defects. Substantial advancements in bone tissue engineering have been achieved in recent years, demonstrating considerable potential for biomedical applications [1]. Scaffolds, essential components of scaffold-based tissue engineering, function as temporary structural frameworks that support cell adhesion, growth, and proliferation until the functional integrity of the host tissue is restored [2]. Extensive research has been conducted regarding tissue-engineering scaffolds composed of metals (e.g., titanium alloys), polymers (e.g., polycaprolactone), hydrogels, and ceramics (e.g., zirconia, alumina, bioglass, and calcium phosphate [CP]) to develop environments conducive to bone regeneration [3,4,5]. The selection of suitable biomaterials requires careful evaluation of their bioactivity, physicochemical characteristics, biodegradability, mechanical properties, and compatibility with additive manufacturing techniques [6].

CP has been widely used in clinical applications for bone repair due to its biochemical composition, which closely resembles the composition of bone. However, its limited mechanical strength hinders its inclusion in load-bearing applications [7]. Additionally, its low biodegradability, which impairs bone healing, restricts its effectiveness in bone regeneration. Recently, research has been conducted to develop a biodegradable metal material that can be degraded in vivo using highly reactive-active metals and has sufficient strength. Among the biodegradable metals being studied is magnesium (Mg), an inorganic component of the human body that is found in bone tissue and does not induce inflammatory reactions or toxicity. Moreover, Mg’s elastic modulus is significantly lower than that of other metals, showing an elastic modulus most similar to that of bone, offering the great advantage of preventing the stress-shielding phenomenon, which is one of the major reasons for the failure of metal implant procedures. However, pure magnesium releases a lot of hydrogen gas (H_2_) upon degradation and has been found to rapidly corrode in aqueous solutions containing chlorine ions (Cl-) present in the human body [8].

The incorporation of magnesium phosphate (MP) into CP has been proposed as a potential solution to these limitations because MP exhibits superior mechanical properties and a higher degradation rate compared to CP [9,10]. MP also demonstrates biocompatibility and supports healing without inducing connective tissue formation [11]. Furthermore, Mg^2+^ promotes osteoblast mineralization, influences intracellular signaling pathways, and serves as a critical cofactor in nucleic acid and protein synthesis [11]. 

The treatment of craniofacial bone defects requires advanced strategies to restore aesthetics and function. Appropriate methods must be used for effective bone defect reconstruction. In this regard, 3D printing is the best way to achieve personalized manufacturing, which can offer advantages such as predesigned architecture and easy processing that can align with the specific shape, size, and porosity of bone tissue [12]. The 3D-printing process eliminates the need for cutting tools or molds, resulting in a high utilization rate of raw materials. This can significantly reduce material waste and shorten production cycles compared to traditional methods [13]. Various additive manufacturing (AM) techniques, such as stereolithography (SLA), fused deposition modeling (FDM) biological 3D printing, 3D powder printing, and DLP 3D printing, are used for bioceramic fabrication in tissue engineering [14,15,16]. To ensure optimal implant integration, MCP samples were prepared using DLP 3D printing in this study. In DLP 3D printing, 3D structures are constructed by solidifying liquid photosensitive resin [17]. It also boasts fast printing speeds with high resolution, which allows the creation of detailed and complex structures. DLP 3D printing minimizes material waste due to its precise layer-by-layer curing process. Moreover, it is particularly well suited for medical applications because it is able to produce biocompatible and degradable materials, which is critical for implants, drug delivery systems, and artificial tissues [18,19].

The objective of this study was to investigate the properties of MCP materials with five different molar ratios, customized using bottom-up DLP 3D-printing technology. The cure depth and rheological properties of MCP slurries were analyzed to ensure successful 3D printing, and thermogravimetric differential thermal analysis (TG-DTA) of the MCP green body was conducted to assess debinding. Finally, the effects of various MCP ratios on mechanical properties, degradation rate, and biocompatibility were evaluated. 

## 2. Materials and Methods

### 2.1. Powder Preparation

Magnesium phosphate octahydrate (Mg_3_(PO_4_)_2_·8H_2_O, purity 95%, Junsei Chemie Inc., Tokyo, Japan) was sintered at 800 °C for 3 h in a furnace (EX-6100, DUO-TRON, Goyang, Republic of Korea). The sintered material was then ball-milled in ethanol for 2 h using a planetary ball mill (Pulverisette 6, Fritsch, Idar-Oberstein, Germany). After the milling process, the sintered cake was manually crushed and sieved through a 53 μm mesh to obtain MP.

### 2.2. Fabrication of MCP Photopolymer Suspensions

MCP suspensions were prepared by mixing MP with calcium phosphate powder (Ca_3_(PO_4_)_2_, purity 98%, Junsei Chemie Inc., Tokyo, Japan) and adding a photopolymerizable binder, which consisted of photocurable rigid resin (XYZ Printing, Inc., Suzhou, China) and acrylate binders (1,6-hexanediol diacrylate [HDDA], Sigma-Aldrich, St. Louis, MO, USA). To enhance photopolymerization and achieve high-quality 3D printing, photoinitiators (Irgacure 819, Ciba Specialty Chemicals, Inc., Basel, Switzerland) were included. These photoinitiators generate active initiating species, such as free radicals, when excited by photons [20]. A dispersant (BYK-180, BYK Chemie GmbH, Wesel, Germany) was added to improve the stability of the suspensions, reduce interparticle attractive forces, and prevent agglomeration [21]. The specimens were divided into five groups based on MP and CP compositions with various molar ratios (4:0, 3:1, 1:1, 1:3, and 0:4) at a 45% volume fraction (Table 1). A planetary centrifugal mixer (ARV-310, Thinky Corp., Tokyo, Japan) was used under vacuum for 10 min to ensure homogeneity and prevent air inclusion before 3D printing.

### 2.3. Rheological Measurement

Flow behaviors of the suspensions were analyzed using a viscometer (FIRST PRODIG CP-1000, LAMY RHEOLOGY, Champagne au Mont d’Or, France). Shear viscosity, which indicates flow resistance, was measured as a function of shear rate. Tests were conducted using a 40 mm diameter plate geometry over a shear rate range of 14.4–100 s^−1^ at room temperature.

### 2.4. Measurement of Cure Depth

A proper cure depth is crucial to prevent incomplete sample curing. The cure depths of MCP suspensions were determined using an optical microscope (EGVM-452 M, iMegascope, Seoul, Republic of Korea) under ultraviolet (UV) light emitted by the DLP 3D printer, with an exposure time of 2–5 s.

### 2.5. Additive Manufacturing

**Figure 1:**
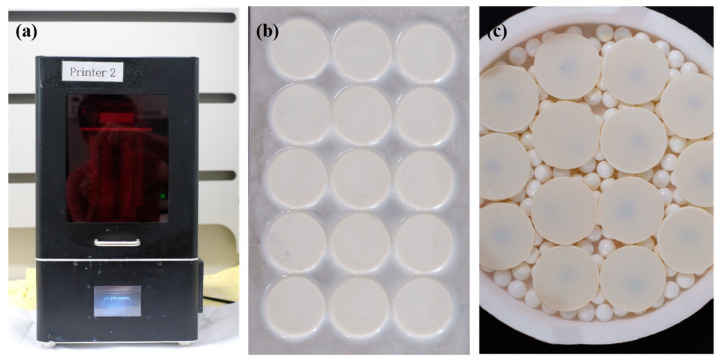
DLP 3D printer and printed MCP samples: (**a**) DLP 3D printer, (**b**) printed disc samples, and (**c**) cleaned samples.

A DLP 3D printer (Phrozen Shuffle, Phrozen Technology, Hsinchu, China) with a bottom-up mechanism was used to cure the suspension with UV light at a wavelength of 405 nm (resolution = 47 µm) (Figure 1a). The UV light was applied for 4 s, passing through a film and curing a layer of suspension onto the build plate (120 mm × 68 mm × 200 mm). The layer thickness was set to 50 µm, and print files (diameter = 18 mm; thickness = 1.8 mm) were designed in the standard triangle language (STL) file format using Autodesk Fusion software (Autodesk Inc., San Francisco, CA, USA). The uncured suspension was removed with alcohol (Figure 1b); samples were dried and cured again for 60 s (Figure 1c).

### 2.6. Thermal Analysis Method

Thermal analysis for debinding, intended to remove organic components, was performed using a TG-DTA system (DTG-60H, Shimadzu, Japan). The samples were heated from 20 °C to 1000 °C under a nitrogen atmosphere with a constant flow rate of 50 mL/min. The final sintering schedule was developed based on the results of thermal analysis. 

### 2.7. Characterization of Samples

#### 2.7.1. Density and Shrinkage

Density was measured using the Archimedes method (ISO-18754:2020). The linear shrinkage of each sample group was assessed by measuring sample lengths before and after sintering (*n* = 10), according to ASTM C326 [22], using Equation (1):

Shrinkage (%) = ((A0 − A△)/A0) × 100(1)

where A0 and A△ represent the areas before and after sintering, respectively.

#### 2.7.2. Microstructural and X-Ray Diffraction (XRD) Analyses

The microstructures of sintered samples from five groups were observed using field-emission scanning electron microscopy (FE-SEM) (JSM-7500F, JEOL, Akishima, Japan) with a conductive layer of platinum. The phase compositions of the specimens were analyzed via XRD (PW-1800, Philips, Amsterdam, The Netherlands). Measurements were conducted using Cu-Kα radiation over a 2θ range of 10–60° at a scanning rate of 5°/min.

#### 2.7.3. Biaxial Flexural Strength Measurement

Specimens (n = 10) were polished to ensure they had smooth surfaces before biaxial flexural strength testing. A universal testing machine (RB 302 ML, R&B Inc., Daejeon, Republic of Korea) was used at a crosshead speed of 0.5 mm/min, with strength calculated according to ISO 6872 (MPa).

#### 2.7.4. Degradation Analysis

The degradation of sintered samples (n = 5) was assessed by immersing them in phosphate-buffered saline (PBS, pH 7.4) at 37 °C in a shaking incubator (NB-205, N-BIOTEK Co., Ltd., Bucheon, Republic of Korea) set at 100 rpm. PBS was refreshed weekly, and sample weights were recorded. Degradation was determined according to weight loss using Equation (2):

Weight loss rate = (W_0_ − W_t_)/W_0_ × 100% (2)

where W_0_ is the initial dry weight, and W_t_ is the dry weight after degradation at a specific time.

### 2.8. In Vitro Cell Experiments

To evaluate cellular proliferation on MCP samples, the bioactivity of 3D-printed specimens (diameter = 6 mm; thickness = 2 mm) was assessed by seeding MG-63 preosteoblast cells on these samples. The MCP samples were cleaned via sonication in ethanol and then sterilized with ethylene oxide gas in an autoclave for 9 h.

MG-63 cells (KCLB 21427, Seoul, Korea Cell Line Bank, Seoul, Republic of Korea) were cultured in Dulbecco’s modified Eagle medium (DMEM) supplemented with 1% penicillin/streptomycin and 10% fetal bovine serum. When MG-63 preosteoblast cells reached confluence, they were collected using TrypLE Express (Gibco, New York, NY, USA). The supernatant was discarded, and 3 mL of fresh culture medium was added. The cell suspension was gently mixed with a pipette tip until it was uniform and transferred to cell culture flasks (BIOFIL, Guangzhou Jet BIOS-Filtration Co., Guangzhou, China). The flasks were incubated at 37 °C with 5% CO_2_, and the medium was replaced every 2–3 days. MG-63 cells at passage 4 were used for cell experiments.

#### 2.8.1. Cell Proliferation

Cell proliferation was evaluated on days 1, 3, and 7 using the XTT assay (EZ-Cytox, DoGenBio Co., Ltd., Seoul, Republic of Korea). Samples (n = 5) were rinsed with PBS, and fresh culture medium was added to each sample. Water-soluble tetrazolium 8 (WST-8) assay solution was then added to each well. After incubation for 30 min, absorbance at 450 nm was recorded using a microplate reader (Synergy H1 Hybrid Multi-Mode Reader, BioTek, Winooski, VT, USA).

#### 2.8.2. Cell Differentiation

Alkaline phosphatase (ALP) activity was measured using an ALP assay kit (Abcam, Cambridge, UK). MG-63 cells were seeded at a density of 2 × 10^4^ cells/well on samples (n = 5) and cultured in osteogenic medium containing DMEM, 1% penicillin/streptomycin, 10% fetal bovine serum, 50 μg/mL of ascorbic acid, 10 mM β-glycerophosphate, and 10 nM dexamethasone. After 7 and 14 days, 1% Triton X-100 solution (Sigma-Aldrich, St. Louis, MO, USA) was added to each well. To achieve complete cell lysis, samples were subjected to three freeze–thaw cycles at −80 °C and room temperature. Lysates (80 μL) were combined with 50 μL of 5 mM p-nitrophenyl phosphate (p-NPP) and incubated at 37 °C for 1 h. The reaction was terminated with 20 μL of stop solution, and absorbance at 405 nm was recorded to determine ALP activity.

### 2.9. Statistical Analysis

Data analysis was performed using SPSS software (v22.0, IBM Corp., New York, NY, USA), and results were reported as means ± standard deviations. The Shapiro–Wilk test was utilized to confirm normality for all groups (*p* > 0.05). Statistical assessments were conducted using one-way analysis of variance and the Tukey test (Origin 7G, OriginLab Corp., Northampton, MA, USA). The threshold for statistical significance was set at *p* < 0.05 (*).

## 3. Results

### 3.1. Characterization of Powder

#### 3.1.1. SEM Analysis

**Figure 2:**
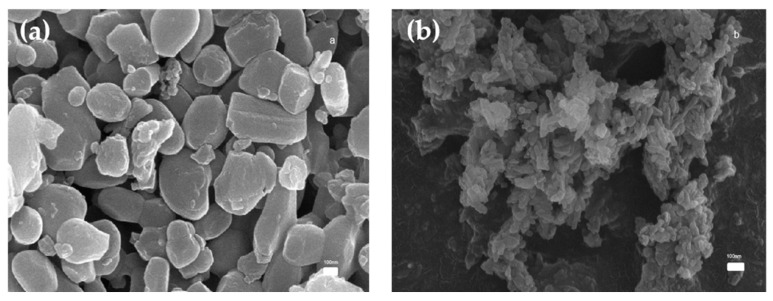
SEM images of (**a**) magnesium phosphate and (**b**) calcium phosphate (50,000×).

Figure 2a,b present SEM images of MP and CP powder particles, respectively. The MP particles exhibit a block-shaped morphology and a diameter of approximately 300 nm. In contrast, the CP particles display a smaller, strip-shaped morphology.

#### 3.1.2. Zeta Potential

**Figure 3:**
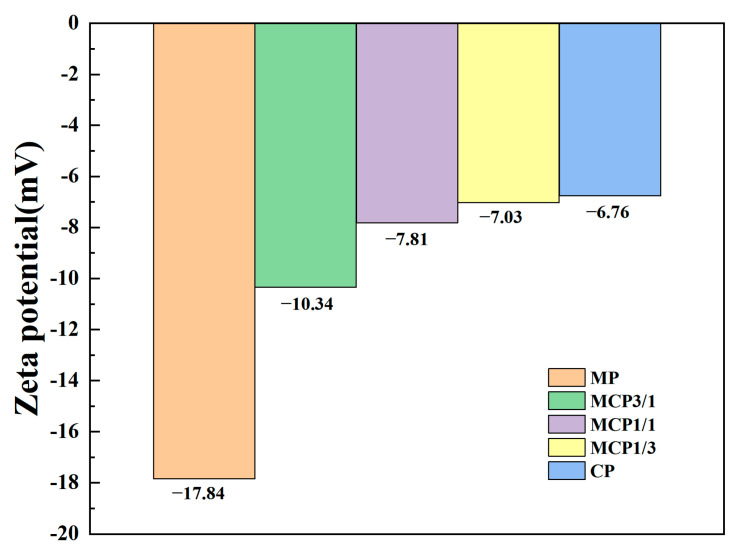
Zeta potential of each group.

A dynamic surface potential (i.e., zeta potential) exists when particles are suspended. In 3D printing processes utilizing the DLP technique, effective deflocculation and well-dispersed solids are essential to enhance print quality [23]. The zeta potential values for MP, MCP3/1, MCP1/1, MCP1/3, and CP were −17.84 mV, −10.34 mV, −7.81 mV, −7.03 mV, and −6.7 mV, respectively (Figure 3).

Negative zeta potential values indicate the presence of numerous negatively charged groups on particle surfaces. The results of a previous study suggested that a negative surface charge benefits bone regeneration applications by promoting cell activity and facilitating the fixation of calcium ions, ultimately accelerating cell growth [24].

### 3.2. Characterization of Suspensions

#### 3.2.1. Viscosity

**Figure 4:**
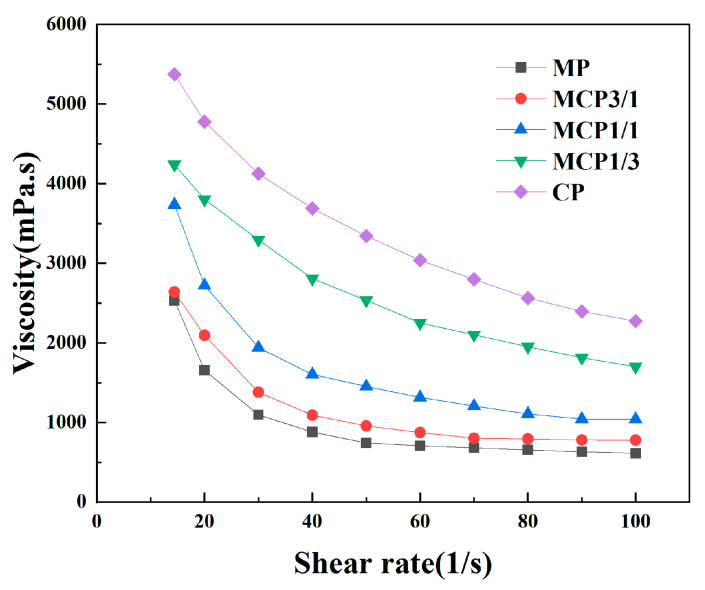
Rheological behavior of suspensions.

SEM analysis revealed that the MP group powder exhibited a larger particle size compared with the CP group powder. A smaller particle size enhances interactions between particles, which can lead to agglomeration and increased viscosity [23]. In this study, the maximum volume fraction used for printing was 45 vol% because a CP volume fraction of 50 vol% resulted in extensive agglomeration, preventing the formation of a stable suspension. Figure 4 shows that the viscosity of the MP suspension was the lowest, whereas the viscosity of the CP suspension was the highest (5373 ± 114.25 m·Pa/s). Particle size influences flow and compaction properties because smaller particles dissolve more rapidly and increase viscosity [25]. The viscosity of these suspensions increased as the volume fraction of the CP particles increased.

#### 3.2.2. Cure Depth

**Figure 5:**
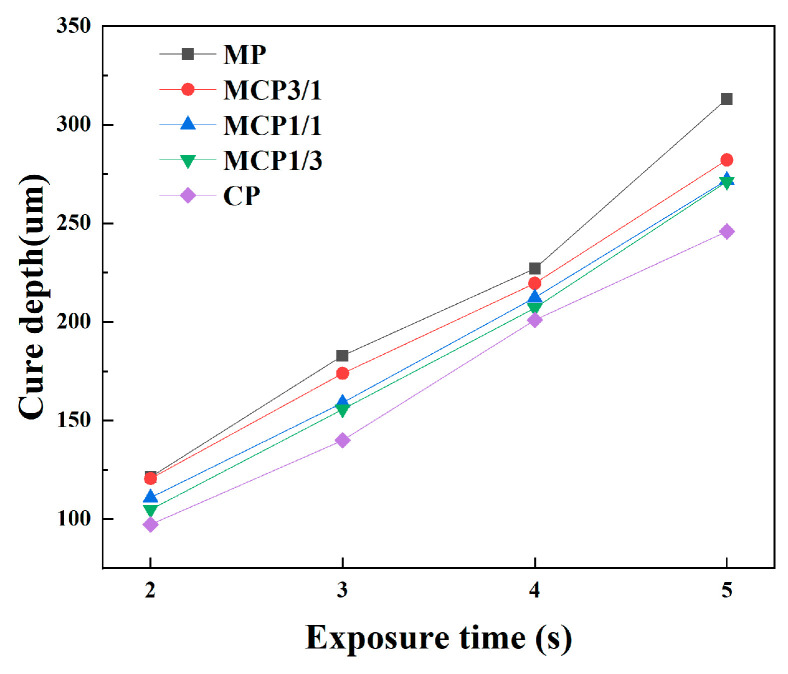
Cure depths of suspensions.

Cure depth is influenced by UV energy and exposure time [26]. To ensure adequate bonding between cured layers, the cure depths of the slurries were evaluated by exposing each group to UV light for 2–5 s (Figure 5). The results indicated that cure depth decreased as exposure time decreased. After 2, 3, 4, and 5 s of photocuring, all the specimens were completely cured. The maximum cure depth, observed after 5 s of photocuring, was 300.02 ± 6.63 µm in the MP group.

### 3.3. Thermal Analysis

**Figure 6:**
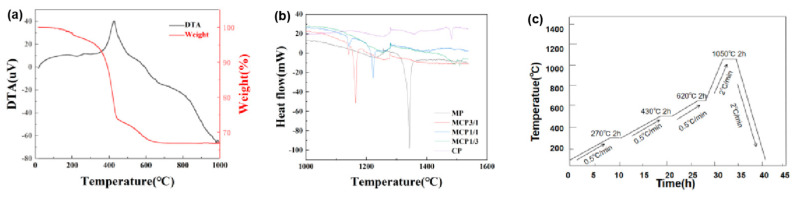
(**a**) TG-DTA results, (**b**) differential scanning calorimetry results, and (**c**) sintering schedule.

Printed samples require debinding to remove inert, non-bioactive, and non-biodegradable organic resins [27]. The debinding behavior of the printed samples was thermally analyzed. Figure 6a shows that exothermic peaks and weight loss at 270 °C, 430 °C, and 620 °C indicated the gradual removal of organic components. These data were used to design a debinding process that produces defect-free parts. To prevent defects within the specimens, the debinding temperatures were set to 270 °C, 430 °C, and 620 °C [28]. The green body was gradually heated at a rate of 0.5 °C/min to prevent defects caused by the evaporation of organic components and avoid pressure buildup that could cause cracks or blistering [29]. To ensure the complete removal of organic components, the temperature was maintained for 2 h at each stage. Figure 6b shows that MCP3/1 had the lowest melting point (1126 °C), as determined via differential scanning calorimetry (DSC). The final sintering step was performed at 1050 °C to prevent sample melting. The sintering schedule is shown in Figure 6c. 

### 3.4. Microstructural Analysis

**Figure 7:**
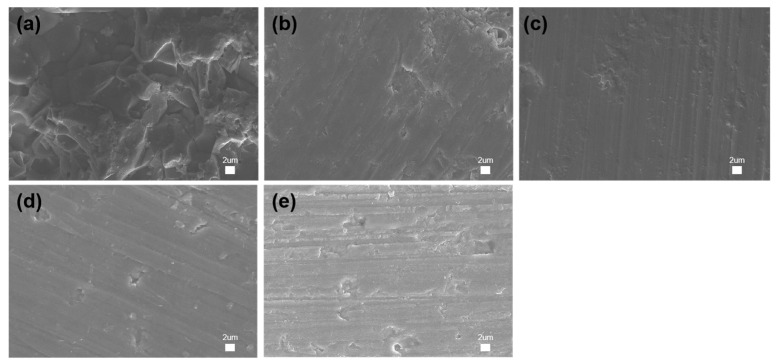
FE-SEM images of the specimens: (**a**) MP, (**b**) MCP3/1, (**c**) MCP1/1, (**d**) MCP1/3, and (**e**) CP (2000×). The bar means 2 µm.

The surface microstructure of each ceramic sample group after sintering was examined using FE-SEM. The MP group exhibited a surface predominantly composed of closely packed rhombohedral crystals (Figure 7a). The MCP3/1 groups displayed a microporous surface structure (Figure 7b). The MCP1/1 group exhibited particles that were fused together and tightly arranged, resulting in a smoother surface texture. A homogeneous surface texture was observed. There was no resin between the powders, indicating that the resin had been removed after sintering and thus formed a dense structure, attributed to the increased shrinkage observed in MCP1/1 (Figure 7c). MCP1/3 and CP groups displayed a microporous surface structure similar to MCP 3/1 group (Figure 7d,e).

### 3.5. XRD

**Figure 8:**
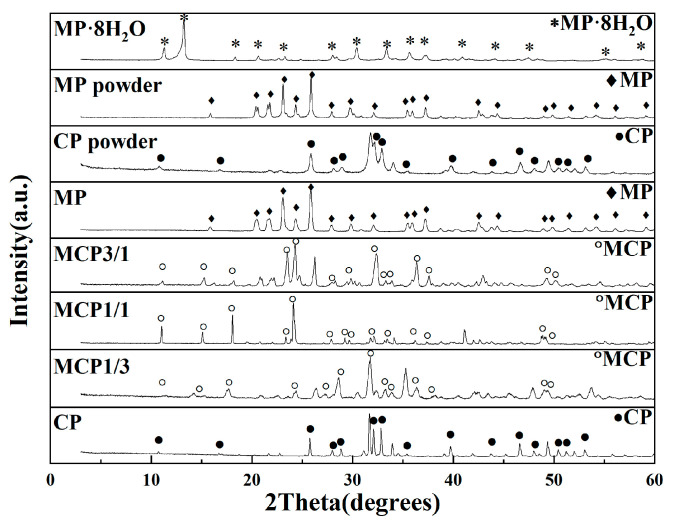
XRD patterns of powders and sintered samples.

Figure 8 presents XRD spectra of Mg_3_(PO_4_)_2_·8H_2_O powder, Mg_3_(PO_4_)_2_ powder, Ca_3_(PO_4_)_2_ powder, and the sintered MP, MCP3/1, MCP1/1, MCP1/3, and CP samples. As shown in Figure 8, all the diffraction detected peaks corresponded to magnesium phosphate octahydrate (Mg_3_(PO_4_)_2_·8H_2_O) (ICOD-01-084-1148), MP (ICOD-00-033-0876), MCP (ICOD-00-11-0231), and CP (Ca_3_(PO_4_)_2_) (ICOD-00-009-0169), which appeared in the diffraction patterns of both the powder and sintered samples. The symbols in the diffraction patterns denote specific phases: asterisks (*) indicate magnesium phosphate octahydrate, diamonds (♦) represent MP, open circles (o) correspond to MCP, and filled circles (●) signify CP. MCP3/1 and MCP1/1 exhibited strong peaks at 23–24°, and MCP1/3 displayed strong peaks at 32–33°, which may have been due to CP residue, and MP was not detected in MCP1/3.

### 3.6. Linear Shrinkage Rate and Density

**Figure 9:**
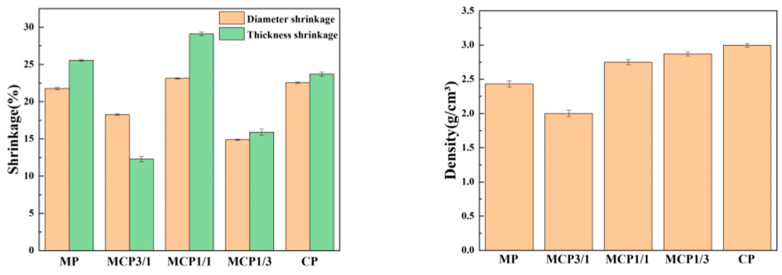
Shrinkage and density properties of MCP samples.

The linear shrinkage and density values measured for each sample group are shown in Figure 9. Linear shrinkage was evaluated in terms of both diameter and thickness. The MCP1/1 samples exhibited the highest linear shrinkage, with values of 23.13 ± 0.007% for diameter and 29.11 ± 0.026% for thickness. The anisotropic nature of linear shrinkage has been reported in previous studies [30]. The CP group exhibited the highest density, at 2.997 ± 0.026 g/cm^3^. However, the MCP3/1 group demonstrated a lower density, likely due to its reduced shrinkage in terms of thickness. 

### 3.7. Biaxial Flexural Strength

**Figure 10:**
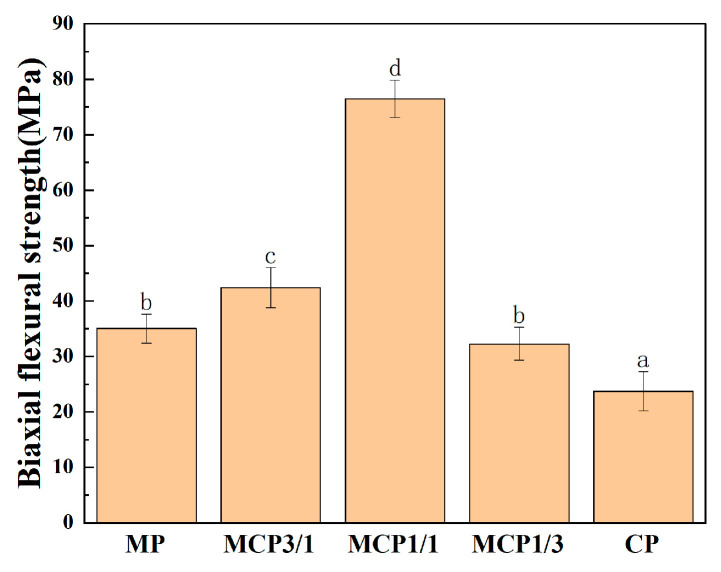
Biaxial flexural strengths of MCP samples. Different letters represent significant differences (*p* < 0.05).

The mechanical properties of the DLP 3D-printed samples were evaluated through uniaxial compressive strength analysis. Figure 10 presents the compressive strength results for each group. Among the groups, MCP1/1 exhibited the highest biaxial flexural strength (76.44 ± 2.81 MPa).

### 3.8. Degradation

**Figure 11:**
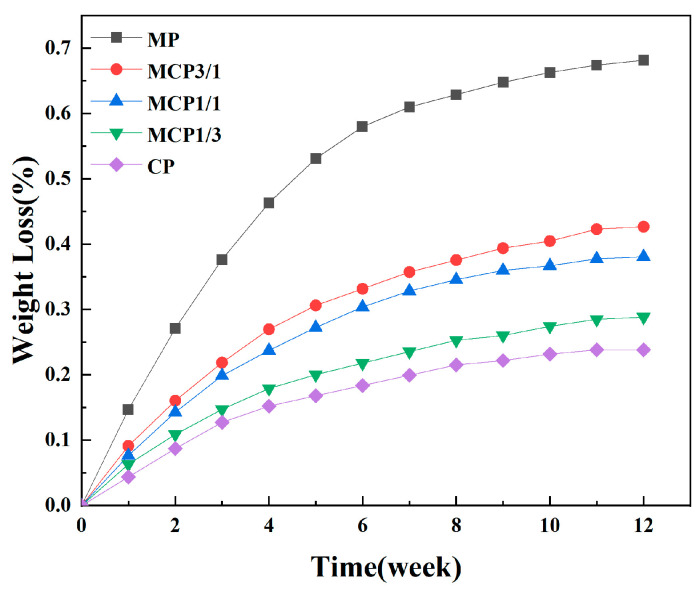
Degradation rate of MCP samples.

Figure 11 illustrates the weight loss ratios of each sample group after their immersion in PBS over various time periods. Sample degradation rates were determined on the basis of weight loss ratios. Among the groups, MP exhibited the most rapid degradation. A comparison of the MCP3/1, MCP1/1, and MCP1/3 samples, which contained varying proportions of MP, indicated that a higher percentage of MP was linked to an accelerated degradation rate. These results are consistent with previous studies, confirming that the degradation rate of MCP increases according to MP percentage [7].

### 3.9. Cell Experiments

**Figure 12:**
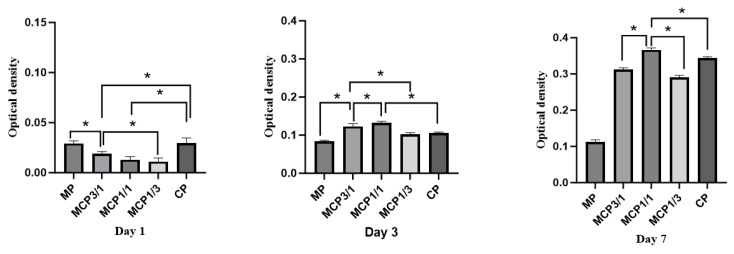
Optical densities of MG-63 cells grown on each sample group. * *p* < 0.05.

Figure 12 presents the cell proliferation results for the MP, MCP3/1, MCP1/1, MCP1/3, and CP groups on days 1, 3, and 7 after MG-63 cell seeding. Cell densities increased over time in all groups. After 4 and 7 days of culturing, the MCP1/1 group exhibited significantly greater cell proliferation compared with the other groups.

**Figure 13:**
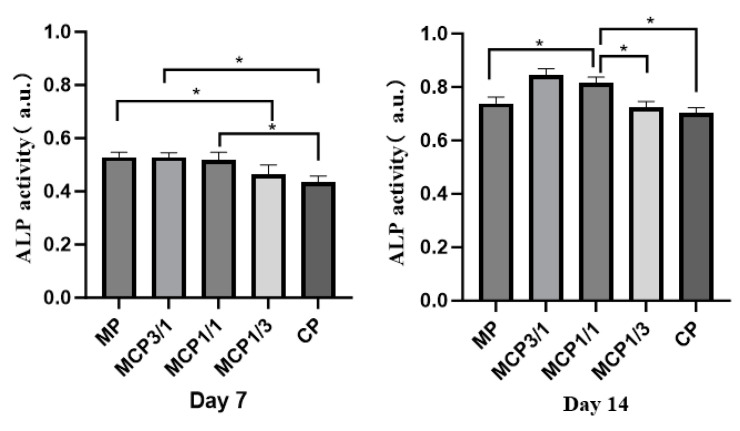
ALP activities in MG-63 cells grown on each sample group. * *p* < 0.05.

ALP analysis was conducted to evaluate cell differentiation. Figure 13 shows cell differentiation at 7 and 14 days after the MG-63 cells were seeded on the MCP samples. The results indicate that ALP activities were higher in the MCP3/1 and MCP1/1 groups than in the CP group at 14 days, suggesting that MCP1/1 enhanced cell differentiation. 

## 4. Discussion

In this study, ceramic samples were fabricated using DLP. MP and CP ceramic powders were incorporated into a binder, which, upon exposure to UV light, initiated polymerization reactions within the resin matrix; this process encapsulated ceramic particles within the binder composite during printing [31]. Thermal treatment was required to remove excess support structure material [32]. Finally, pure ceramic samples were obtained.

We used a bottom-up DLP additive manufacturing technique to create ceramic components. “Bottom-up” and “top-down”, the two main DLP approaches, exhibit slight differences [33]. A bottom-up approach enables precise and consistent layer thickness while requiring only a small amount of photosensitive paste. This low paste requirement reduces the start-up cost of bottom-up DLP 3D printers relative to top-down printers. Therefore, bottom-up DLP 3D printers are more suitable for small and medium-sized enterprises as well as research institutions.

We used a dispersant to prepare a ceramic slurry. The phosphate group in the dispersant exerts an anchoring effect, facilitating its combination with ceramic particles; thus, it produces a stable and well-dispersed ceramic slurry. Additionally, the polymer chain at the other end of the dispersant is compatible with non-aqueous media, forming a thicker layer on the surfaces of ceramic particles. This layer generates sufficient repulsive forces between particles, thereby enhancing dispersibility [34]. A homogeneous ceramic slurry with a high solid loading and low viscosity is essential for the fabrication of high-precision and high-quality ceramic scaffolds [35]. A high solid loading in ceramic powders minimizes shrinkage and enhances sample density after thermal treatment, reducing the likelihood of cracks during debinding and sintering [36]. This management of cracking ensures that the final parts achieve high density and facilitates a smooth recoating process during DLP 3D printing [37].

Borlaf et al. [38] noted that even when cure depth exceeds layer thickness, strong bonding between cured layers cannot always be achieved. Insufficient exposure time can result in poor bonding, leading to crack formation during the debinding process, which affects the properties of the final samples. Conversely, excessive exposure time may partially cure the slurry outside the intended printing area due to scattered UV light, adversely affecting the dimensional accuracy of the green parts [39]. Therefore, balancing accuracy and exposure time are crucial in order to obtain optimal 3D-printing outcomes. Moreover, Jacobs et al. proposed that the appropriate layer thickness for photopolymerization-based 3D printing is approximately one-fourth of the polymerization depth [40].

Shrinkage rates were lower in the diameter direction than in the thickness direction. During the printing process, each cured layer separated from the film in the thickness direction after curing, resulting in relatively less compact bonding between layers in this direction. During the subsequent heating process, gaps between layers in the thickness direction gradually decreased due to the combined effects of thermal treatment and gravity. Consequently, shrinkage rates were higher in the thickness direction than in the diameter direction [41].

The compressive strength of the composite samples was highest when MP and CP were combined at a molar ratio of 1:1. This group exhibited superior compressive strength compared with the other groups, primarily due to its high shrinkage rate and increased densification. This increased densification may be related to the use of a sintering temperature near the melting point of MP. During sintering, the liquid-phase MP enveloped the CP particles, promoting better particle packing and greater sample densification.

Ideally, biomaterials should dynamically interact with cells and stimulate their growth [42]. Osteoblasts play a central role in bone formation and are crucial for bone regeneration; they contribute to the maintenance of a bone-remodeling equilibrium. Adequate osteoblast proliferation and differentiation are essential for proper bone formation and regeneration [43]. Calcium is vital for numerous cellular functions, including proliferation, differentiation, and apoptosis [44]. Similarly, magnesium stimulates intracellular signaling pathways and accelerates bone regeneration [45,46].

MG-63 cells exhibited the ability to proliferate on MCP, as demonstrated by the XTT assay, indicating a positive cellular response to this material. Notably, the proliferation rate was higher on MCP1/1 than on CP, suggesting that MCP1/1 more effectively promotes cellular proliferation. In addition to proliferation, the capacity of cells to differentiate on materials reveals their viability and biocompatibility. When MG-63 cells were cultured on MCP1/1, cell proliferation was observed to increase by day 3. However, ALP activity showed a significant increase on day 14, which may be attributed to the early induction of proliferation and the subsequent promotion of differentiation at a slightly later stage. This effect was likely due to the release of magnesium and calcium ions from MCP into the cell culture medium. And continuous dissolution of MCP creates a calcium- and magnesium-enriched environment, which likely plays a role in stimulating both cell proliferation and differentiation [47]. 

## 5. Conclusions

MCP bioceramics were successfully fabricated using DLP additive manufacturing technology. The MP particles exhibited a larger size than the CP particles, and the viscosity of each suspension increased according to the volume fraction of CP particles. The MCP1/1 material, customized via 3D printing technology, demonstrated good mechanical properties and biocompatibility. Additionally, its degradation rate was higher than that of CP. The material exhibited excellent properties and showed strong potential for tissue-engineering applications in the near future.

## Figures and Tables

**Table 1:** Compositions of MCP suspensions in each group.

Group	MCP (vol%) (Mg:CP)	Resin (vol%)	HDDA (vol%)	Photoinitiator (vol%)	Dispersant (vol%)	**Total** **(vol%)**
MP	45% (4:0)	8.52%	37.30%	0.04%	9.14%	100%
MCP3/1	45% (3:1)	8.36%	36.62%	0.04%	9.98%	100%
MCP1/1	45% (1:1)	8.19%	35.86%	0.04%	10.91%	100%
MCP1/3	45% (1:3)	8.00%	35.02%	0.04%	11.94%	100%
CP	45% (0:4)	7.78%	34.09%	0.04%	13.09%	100%

## Data Availability

The original contributions presented in the study are included in the article, further inquiries can be directed to the corresponding author.

## Abbreviations

The following abbreviations are used in this manuscript:

3D	three-dimensional
DLP	digital light processing
MCP	magnesium calcium phosphate
CP	calcium phosphate
MP	magnesium phosphate
AM	additive manufacturing
SLA	stereolithography
FDM	fused deposition modeling
TG-DTA	thermogravimetric–differential thermal analysis
HDDA	1,6-hexanediol diacrylate
UV	ultraviolet
STL	standard triangle language
DMEM	Dulbecco’s modified Eagle medium
PBS	phosphate-buffered saline
WST-8	water-soluble tetrazolium 8
ALP	alkaline phosphatase
DSC	differential scanning calorimetry

## References

[1] Smith B.T., Shum J., Wong M., Mikos A.G., Young S. (2015). Bone Tissue Engineering Challenges in Oral & Maxillofacial Surgery, Engineering Mineralized and Load Bearing Tissues.

[2] Wubneh A., Tsekoura E.K., Ayranci C., Uludağ H. (2018). Current state of fabrication technologies and materials for bone tissue engineering. Acta Biomater..

[3] He J., Li K., Wu T., Chen J., Li S., Zhang X. (2023). Research progress in degradable metal-based multifunctional scaffolds for bone tissue engineering. Med. Comm. Biomater. Appl..

[4] Zare Y., Shabani I. (2016). Polymer/metal nanocomposites for biomedical applications. Mater. Sci. Eng. C.

[5] Ribas R.G., Schatkoski V.M., do Amaral Montanheiro T.L., de Menezes B.R.C., Stegemann C., Gonçalves Leite D.M., Thim G.P. (2019). Current advances in bone tissue engineering concerning ceramic and bioglass scaffolds: A review. Ceram. Int..

[6] Arif Z.U., Khalid M.Y., Noroozi R., Sadeghianmaryan A., Jalalvand M., Hossain M. (2022). Recent advances in 3D-printed polylactide and polycaprolactone-based biomaterials for tissue engineering applications. Int. J. Biol. Macromol..

[7] Wu F., Wei J., Guo H., Chen F., Hong H., Liu C. (2008). Self-setting bioactive calcium–magnesium phosphate cement with high strength and degradability for bone regeneration. Acta Biomater..

[8] Eugen G., Claus M., Anna-Maria S., Niklas D., Philipp S., Andrea E., Andrea M.-L., Elke V. (2023). Degradation of 3D-printed magnesium phosphate ceramics in vitro and a prognosis on their bone regeneration potential. Bioact. Mater..

[9] Klammert U., Ignatius A., Wolfram U., Reuther T., Gbureck U. (2011). In vivo degradation of low temperature calcium and magnesium phosphate ceramics in a heterotopic model. Acta Biomater..

[10] Gelli R., Ridi F. (2023). An Overview of Magnesium-Phosphate-Based Cements as Bone Repair Materials. J. Funct. Biomater..

[11] Zhao Q., Ni Y., Wei H., Duan Y., Chen J., Xiao Q., Gao J., Yu Y., Cui Y., Ouyang S. (2023). Ion incorporation into bone grafting materials. Periodontology.

[12] Quan H., Zhang T., Xu H., Luo S., Nie J., Zhu X. (2020). Photo-curing 3D printing technique and its challenges. Bioact. Mater..

[13] Chen F., Zhu H., Wu J.-M., Chen S., Cheng L.-J., Shi Y.-S., Mo Y.-C., Li C.-H., Xiao J. (2020). Preparation and biological evaluation of ZrO_2_ all-ceramic teeth by DLP technology. Ceram. Int..

[14] Lei B., Gao X., Zhang R., Yi X., Zhou Q. (2022). In situ magnesium phosphate/polycaprolactone 3D-printed scaffold induce bone regeneration in rabbit maxillofacial bone defect model. Mater. Des..

[15] Schaufler C., Schmitt A.-M., Moseke C., Stahlhut P., Geroneit I., Brückner M., Meyer-Lindenberg A., Vorndran E. (2022). Physicochemical degradation of calcium magnesium phosphate (stanfieldite) based bone replacement materials and the effect on their cytocompatibility. Biomed. Mater..

[16] Chen Z., Li Z., Li J., Liu C., Lao C., Fu Y., Liu C., Li Y., Wang P., He Y. (2019). 3D printing of ceramics: A review. J. Eur. Ceram. Soc..

[17] Wang Y., Chen S., Liang H., Liu Y., Bai J., Wang M. (2022). Digital light processing (DLP) of nano biphasic calcium phosphate bioceramic for making bone tissue engineering scaffolds. Ceram. Int..

[18] Zhang J., Hu Q., Wang S., Tao J., Gou M. (2019). Digital light processing based three-dimensional printing for medical applications. Int. J. Bioprinting.

[19] Coppola B., Montanaro L., Palmero P. (2022). DLP Fabrication of Zirconia Scaffolds Coated with HA/β-TCP Layer: Role of Scaffold Architecture on Mechanical and Biological Properties. J. Funct. Biomater..

[20] Bagheri A., Jin J. (2019). Photopolymerization in 3D printing. ACS Appl. Polym. Mater..

[21] Liu M., Wang Y., Zhang H., Liu X., Wei Q., Li M., Liu Z., Bao C., Zhang K. (2023). Effects of dispersant concentration on the properties of hydroxyapatite slurry and scaffold fabricated by digital light processing. J. Manuf. Process..

[22] (1997). Standard Test Method for Drying and Firing Shrinkages of Ceramic Whiteware Clays.

[23] Esteves A.V., Martins M.I., Soares P., Rodrigues M., Lopes M., Santos J. (2022). Additive manufacturing of ceramic alumina/calcium phosphate structures by DLP 3D printing. Mater. Chem. Phys..

[24] Doostmohammadi A., Monshi A., Salehi R., Fathi M., Karbasi S., Pieles U., Daniels A. (2012). Preparation, chemistry and physical properties of bone-derived hydroxyapatite particles having a negative zeta potential. Mater. Chem. Phys..

[25] Jun M.-J., Kang J.-H., Sakthiabirami K., Toopghara S.A.H., Kim Y.-S., Yun K.-D., Park S.-W. (2023). The Impact of Particle Size and Surface Treatment of Zirconia Suspension for Photocuring Additive Manufacturing. Materials.

[26] Wei L., Zhang J., Yu F., Zhang W., Meng X., Yang N., Liu S. (2019). A novel fabrication of yttria-stabilized-zirconia dense electrolyte for solid oxide fuel cells by 3D printing technique. Int. J. Hydrogen Energy.

[27] Scalera F., Corcione C.E., Montagna F., Sannino A., Maffezzoli A. (2014). Development and characterization of UV curable epoxy/hydroxyapatite suspensions for stereolithography applied to bone tissue engineering. Ceram. Int..

[28] Muralithran G., Ramesh S. (2000). The effects of sintering temperature on the properties of hydroxyapatite. Ceram. Int..

[29] Thomas-Vielma P., Cervera A., Levenfeld B., Várez A. (2008). Production of alumina parts by powder injection molding with a binder system based on high density polyethylene. J. Eur. Ceram. Soc..

[30] He R., Liu W., Wu Z., An D., Huang M., Wu H., Jiang Q., Ji X., Wu S., Xie Z. (2018). Fabrication of complex-shaped zirconia ceramic parts via a DLP- stereolithography-based 3D printing method. Ceram. Int..

[31] Zakeri S., Vippola M., Levänen E. (2020). A comprehensive review of the photopolymerization of ceramic resins used in stereolithography. Addit. Manuf..

[32] Sim J.-H., Koo B.-K., Jung M., Kim D.-S. (2022). Study on Debinding and Sintering Processes for Ceramics Fabricated Using Digital Light Processing (DLP) 3D Printing. Processes.

[33] Santoliquido O., Colombo P., Ortona A. (2019). Additive Manufacturing of ceramic components by Digital Light Processing: A comparison between the “bottom-up” and the “top-down” approaches. J. Eur. Ceram. Soc..

[34] Sun J., Binner J., Bai J. (2019). Effect of surface treatment on the dispersion of nano zirconia particles in non-aqueous suspensions for stereolithography. J. Eur. Ceram. Soc..

[35] Zhang K., Xie C., Wang G., He R., Ding G., Wang M., Dai D., Fang D. (2019). High solid loading, low viscosity photosensitive Al_2_O_3_ slurry for stereolithography based additive manufacturing. Ceram. Int..

[36] Zhang K.Q., Meng Q.Y., Zhang X.Q., Qu Z.L., Jing S.K., He R.J. (2021). Roles of solid loading in stereolithography additive manufacturing of ZrO_2_ ceramic. Int. J. Refract. Met. Hard Mater..

[37] Zhang S., Sha N., Zhao Z. (2017). Surface modification of α-Al_2_O_3_ with dicarboxylic acids for the preparation of UV-curable ceramic suspensions. J. Eur. Ceram. Soc..

[38] Borlaf M., Serra-Capdevila A., Colominas C., Graule T. (2019). Development of UV-curable ZrO_2_ slurries for additive manufacturing (LCM-DLP) technology. J. Eur. Ceram. Soc..

[39] Halloran J.W., Tomeckova V., Gentry S., Das S., Cilino P., Yuan D., Guo R., Rudraraju A., Shao P., Wu T. (2011). Photopolymerization of powder suspensions for shaping ceramics. J. Eur. Ceram. Soc..

[40] Jacobs P.F. (1992). Rapid Prototyping & Manufacturing: Fundamentals of Stereolithography.

[41] Ge M., Xie D., Yang Y., Tian Z. (2023). Sintering densification mechanism and mechanical properties of the 3D-printed high-melting-point-difference magnesium oxide/calcium phosphate composite bio-ceramic scaffold. J. Mech. Behav. Biomed. Mater..

[42] Sader M.S., LeGeros R.Z., Soares G.A. (2008). Human osteoblasts adhesion and proliferation on magnesium-substituted tricalcium phosphate dense tablets. J. Mater. Sci. Mater. Med..

[43] Salhotra A., Shah H.N., Levi B., Longaker M.T. (2020). Mechanisms of bone development and repair. Nat. Rev. Mol. Cell Biol..

[44] Patergnani S., Danese A., Bouhamida E., Aguiari G., Previati M., Pinton P., Giorgi C. (2020). Various Aspects of Calcium Signaling in the Regulation of Apoptosis, Autophagy, Cell Proliferation, and Cancer. Int. J. Mol. Sci..

[45] Yoshizawa S., Brown A., Barchowsky A., Sfeir C. (2014). Magnesium ion stimulation of bone marrow stromal cells enhances osteogenic activity, simulating the effect of magnesium alloy degradation. Acta Biomater..

[46] Salamanca E., Pan Y.-H., Sun Y.-S., Hsueh H.-W., Dorj O., Yao W.-L., Lin J.C.-Y., Teng N.-C., Watanabe I., Abe S. (2022). Magnesium Modified β-Tricalcium Phosphate Induces Cell Osteogenic Differentiation In Vitro and Bone Regeneration In Vivo. Int. J. Mol. Sci..

[47] Jia J., Zhou H., Wei J., Jiang X., Hua H., Chen F., Wei S., Shin J.W., Liu C. (2010). Development of magnesium calcium phosphate biocement for bone regeneration. J. R. Soc. Interface.

